# Supplementary material for: Adaptive laboratory evolution of a genome-reduced Escherichia coli
Source: Nat Commun. 2019 Feb 25;10:935. doi: 10.1038/s41467-019-08888-6 (PMC6389913; doi:10.1038/s41467-019-08888-6)
Supplement: Supplementary file 1 — Supplementary Information [file 41467_2019_8888_MOESM1_ESM.pdf]

Supplementary Information for

Adaptive laboratory evolution of a genome-reduced *Escherichia coli*

Choe et al.

This PDF file includes:

Supplementary Figs. 1 to 19
Supplementary Tables 1 to 3
Supplementary References

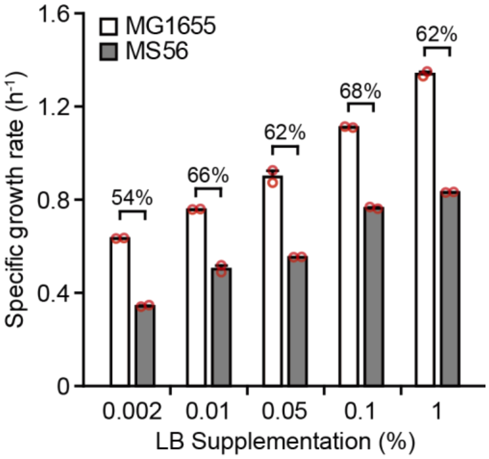

Supplementary Fig. 1. Specific growth rate of MG1655 and MS56 grown in M9 minimal medium supplemented with LB medium. Percentages indicate fraction of MS56 growth rate to that of MG1655. Owing to low growth rate of MS56 on M9 glucose medium, we supplemented a trace amount of LB medium to the M9 glucose medium. LB medium was supplemented with various concentrations. The growth rate of MS56 was recovered as approximately 2/3 of MG1655 when LB concentration was 0.01% or higher. However, the number of cell divisions was low to conduct ALE under 0.05% LB supplementation with regard to culture contamination and speed of adaptation. Thus, 0.1% of LB medium was finally supplemented in the ALE experiment. Error bars indicate s.d. of two biological replicates. Dot plot shows individual data points.

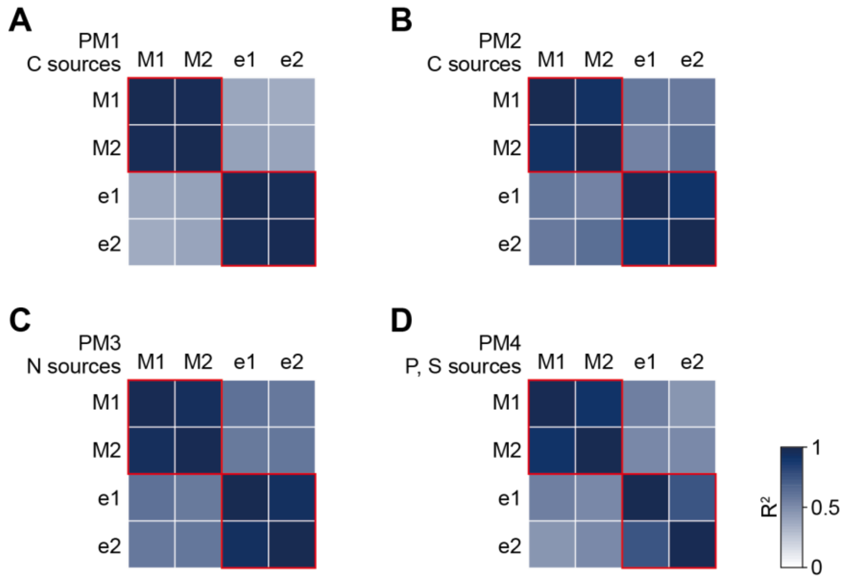

Supplementary Fig. 2. Pearson correlation (R^2) between biological replicates of phenotype microarray. Heatmaps show reproducibility of (A) PM1, (B) PM2, (C) PM3B, and (D) PM4A microplates. M: MG1655, e: eMS57, C: carbon, N: nitrogen, P: phosphorus, S: sulfur. Correlation between duplicates was over 0.76 (with an average of 0.92)

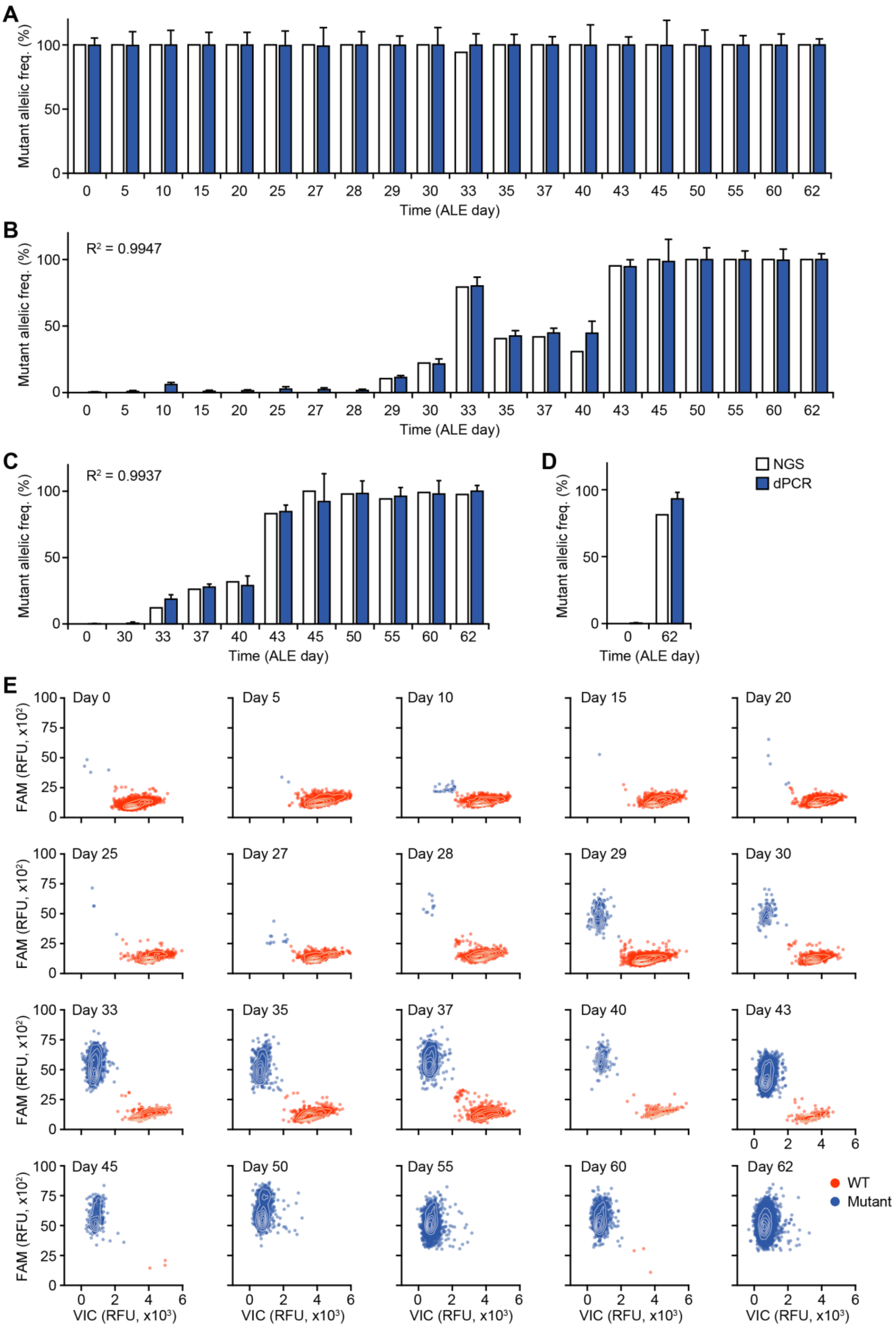
A
Mutant allelic freq. (%)
Time (ALE day)
B
R² = 0.9947
C
R² = 0.9937
D
NGS
dPCR
E
FAM (RFU, x10²)
VIC (RFU, x10³)
Day 0
Day 5
Day 10
Day 15
Day 20
Day 25
Day 27
Day 28
Day 29
Day 30
Day 33
Day 35
Day 37
Day 40
Day 43
Day 45
Day 50
Day 55
Day 60
Day 62
WT
Mutant

Supplementary Fig. 3. Confirmation of variants detected by whole genome sequencing using dPCR-coupled TaqMan assay. Allelic frequencies of sequence variants on (A) *ampD*, (B) *ilvN*, (C) *cspC*, and (D) *yifB* detected from Next Generation sequencing and dPCR had high correlation. Error bar indicates 105% confidence interval. (E) Plots show wild-type and mutant allele of ilvN in during the ALE. Each dot indicates an individual PCR reaction of dPCR. FAM and VIC fluorescence dyes were coupled with probe for mutant and reference DNA sequence, respectively. FAM-high and VIC-low dots (orange) indicate mutant allele, while FAM-low and VIC-high dots (blue) indicate amplification of wildtype allele.

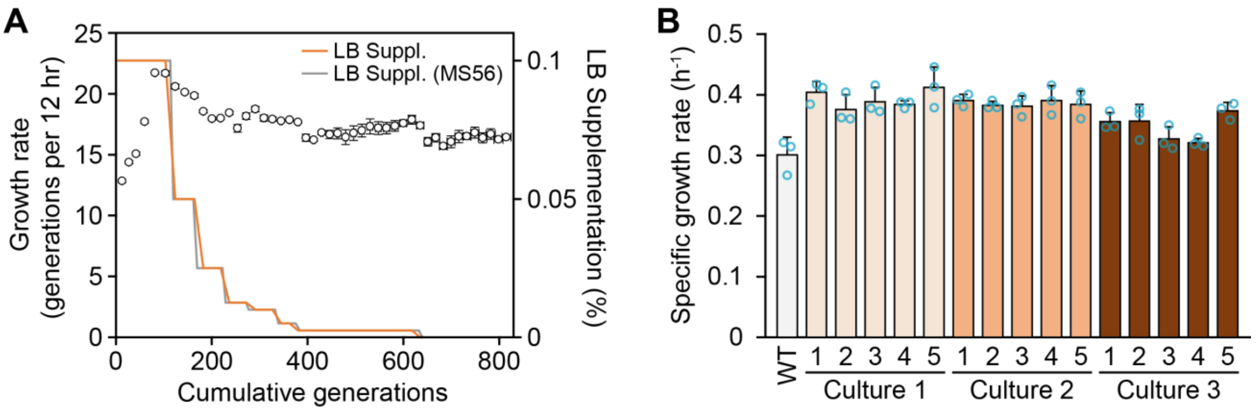

Supplementary Fig. 4. Adaptive laboratory evolution of a wild type *E. coli* MG1655. (A) Growth rate trajectory shows growth rate increase during the ALE and supplementation of LB medium. Amount of LB supplementation was reduced the same as ALE of MS56. Orange line indicates LB supplementation in MG1655 ALE and gray line shows LB supplementation in ALE of MS56 as a reference. (B) Growth rate of 15 clones isolated from three end point cultures of ALE. Error bars indicate s.d. of three biological replicates. Dot plot shows individual data points.

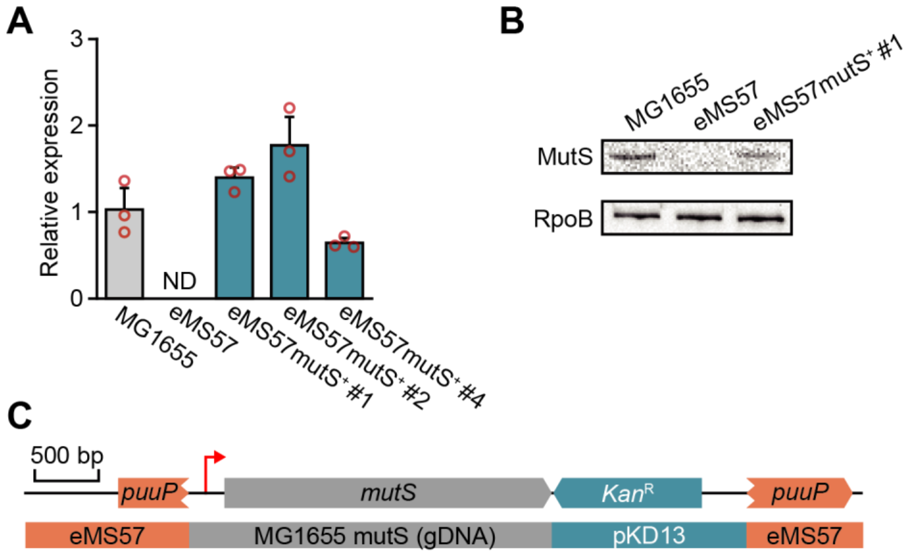

Supplementary Fig. 5. Validation of eMS57mutS^{+} strain. (A) Among three mutS knocked-in clones isolated, clone #1 showed the most similar expression level when compared to MG1655. Clone #1 was termed eMS57mutS+ and used for further experiments. Error bars indicate s.d. of three biological replicates. Dot plot shows individual data points. (B) Production of MutS protein was confirmed by western blot. RpoB was used as a control. (C) Genomic map of *mutS* knocked-in locus in the eMS57mutS^{+} strain.

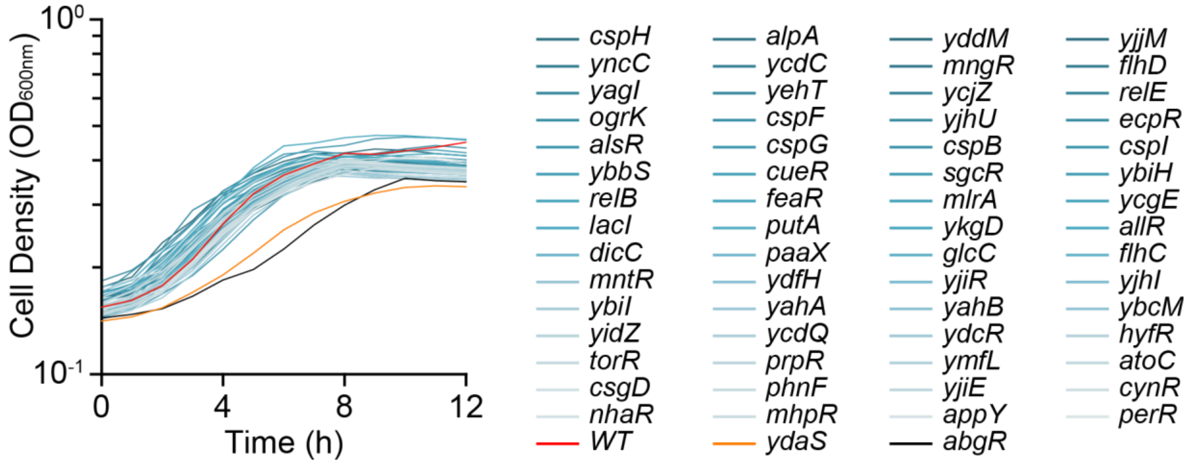

Supplementary Fig. 6. Growth curve of *E. coli* lacking one of the 62 transcription factors. Growth of 62 knock-out strains in M9 glucose medium was monitored in a 96-well plate on a Synergy H1 microplate reader (Bio-Tek). The plate was incubated at 37°C with constant double orbital shaking (5 mm amplitude). WT: *E. coli* K-12 strain BW25113. Deletion strains were obtained from single gene knockout collection (the Keio collection). *dicA* single knockout strain was not tested, because it is not contained in the Keio strain collection as only the *ΔdicA ΔdicB* double knockout strain is viable. No strain showed significant growth retardation in M9 glucose medium, although the growth rates of the *ΔydaS* and *ΔabgR* strains were decreased to 72.4% and 69.8% of that of wild-type *E. coli*, respectively. Growth retardation of *ΔydaS* did not result from *ydaS* deletion[1].

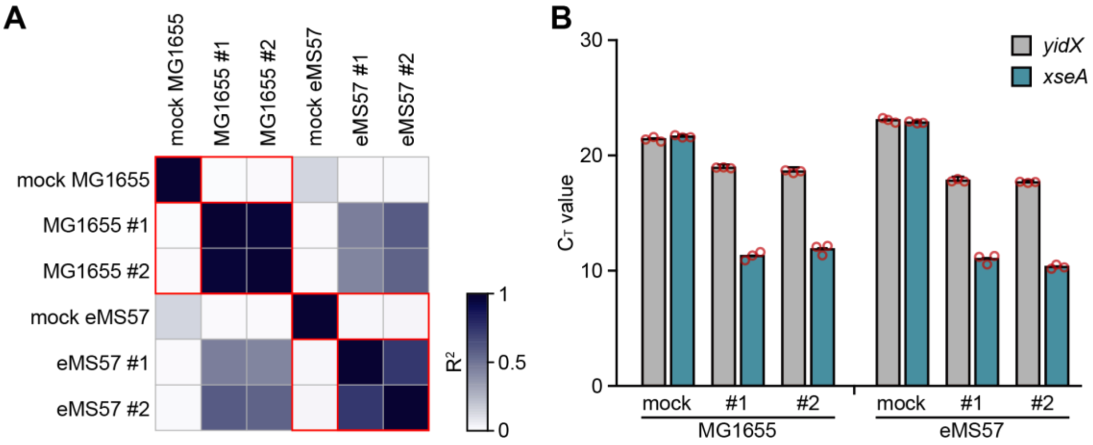

Supplementary Fig. 7. Reproducibility and validation of ChIP-Seq. (A) Pearson correlation (R^2) between biological replicates and mock samples. Reproducibility between ChIP-Seq samples was estimated by extracting intensities of 100,000 genomic positions. Biological replicates had a Pearson correlation (r^2) larger than 0.74 (average of 0.86). As expected, mock samples had a correlation no higher than 0.13 with any sample. (B) ChIP-Seq enrichment was confirmed by qPCR of positive (*xseA*) and negative (*yidX*) genes. Error bars indicate s.d. of three technical replicates. Dot plot shows individual data points.

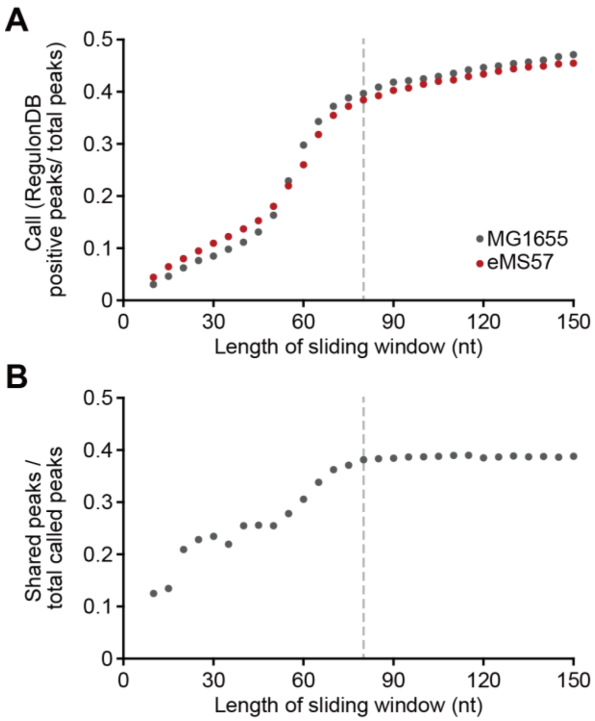

Supplementary Fig. 8. Determination of sliding window size when comparing ChIP-Seq peaks. (A) Number of ChIP-Seq peaks with RegulonDB evidence compared with raw peaks (MG1655: 1,062 peaks and eMS57: 1,089 peaks) varies according to sliding window size. (B) Number of peaks bound by both wild-type and mutant σ^{70} increases as the total number of called peaks increases. However, the ratio of shared peaks to total peaks reaches a plateau at a sliding window of 80 nt or longer.

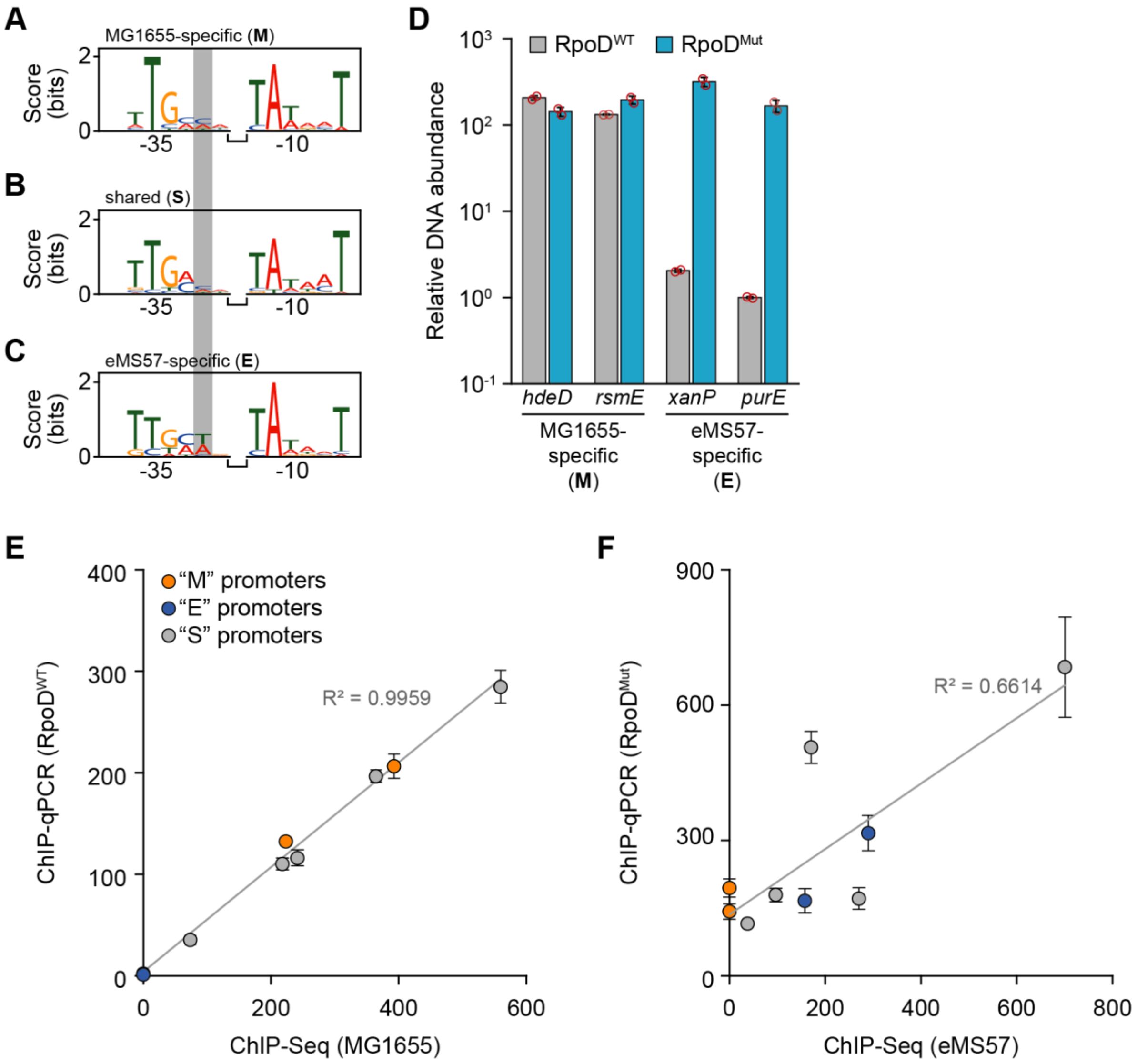

Supplementary Fig. 9. Consensus sequence of promoters used specifically in MG1655 (M), specifically in eMS57 (E), or in both strains (S). (A) MG1655-specific promoters, n = 56. (B) Shared promoters, n = 98. (C) eMS57-specific promoters, n = 320. (D) Native or mutant RpoD was heterologously expressed in MG1655 and bound promoter was immunoprecipitated by c-Myc epitope tagged to RpoD. Binding strength (DNA abundance in immune-precipitated DNA) on M and E promoters are presented. Ser253Pro mutation was sufficient for increasing the specificity to E promoters. However, mutation in RpoD did not change the binding to M promoters. Dot plot shows individual data points. (E) Promoter specificity of native RpoD tested by ChIP-qPCR showed high reproducibility with ChIP-Seq for MG1655. (F) Promoter specificity of mutant RpoD measured by ChIP-qPCR did not correlate with eMS57 ChIP-Seq, although specificity on the E promoters was increased. Binding of M and S promoters in eMS57 appeared to result from the collective interaction between mutant RpoD and other *trans*-acting elements, such as transcription factors. Error bars indicate the s.d. of two biological replicates, each consisting of three technical replicate reactions.

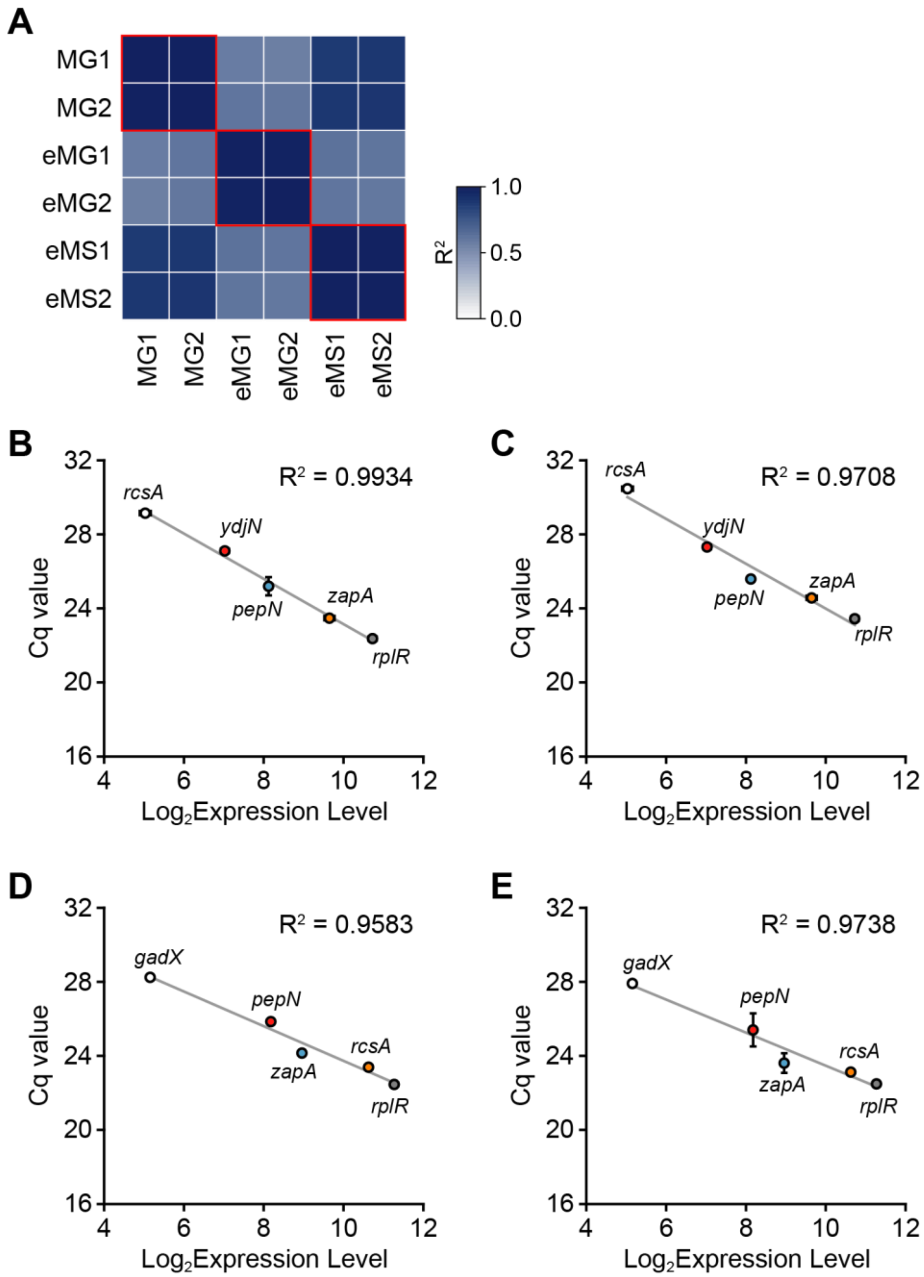

Supplementary Fig. 10. Reproducibility and qRT-PCR validation of RNA-Seq. (A) Heatmap shows Pearson correlation (R^2) of RNA-Seq replicates. The biological replicates had a correlation of 0.996 or higher. (B to D) Correlations between RNA expression level calculated from RNA-Seq and qRT-PCR are plotted. (B) MG1655 Biol. Rep. 1 (C) MG1655 Biol. Rep.2 (D) eMS57 Biol. Rep. 1 (E) eMS57 Biol. Rep. 2. Each biological replicate was tested and qRT-PCR reactions were performed in triplicate. Error bars indicate s.d. of the replicates. Gene names and Pearson correlation (r^2) of Log2 RNA expression level and Cq value are shown on the plot. Expression levels measured by RNA-Seq and qRT-PCR had high correlation ($r^2 > 0.95$).

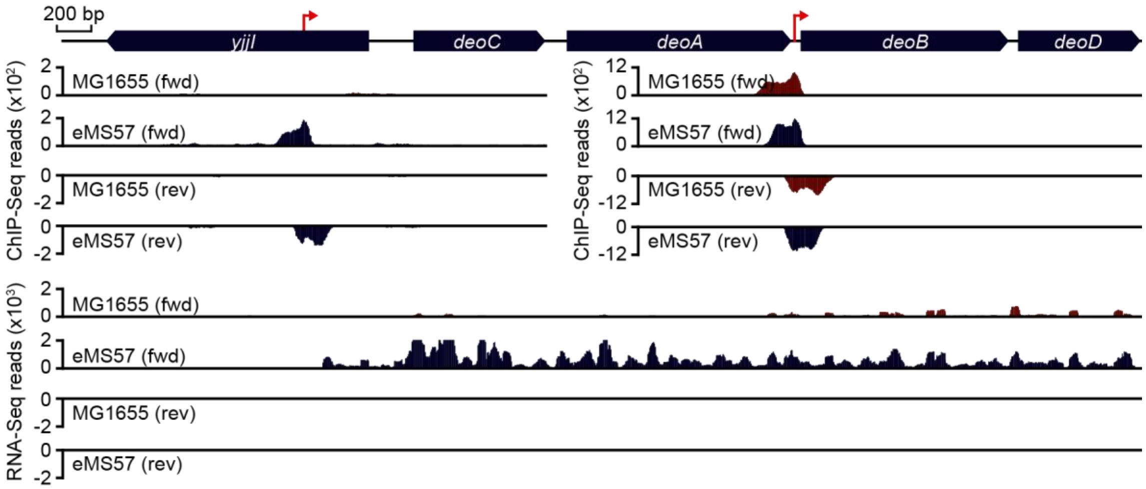

Supplementary Fig. 11. ChIP-Seq and RNA-Seq profiles of *deoCABD*. The results show different σ^{70} binding and transcription of deoxynucleoside degradation operon *deoCABD*. Red arrows indicate operon promoters.

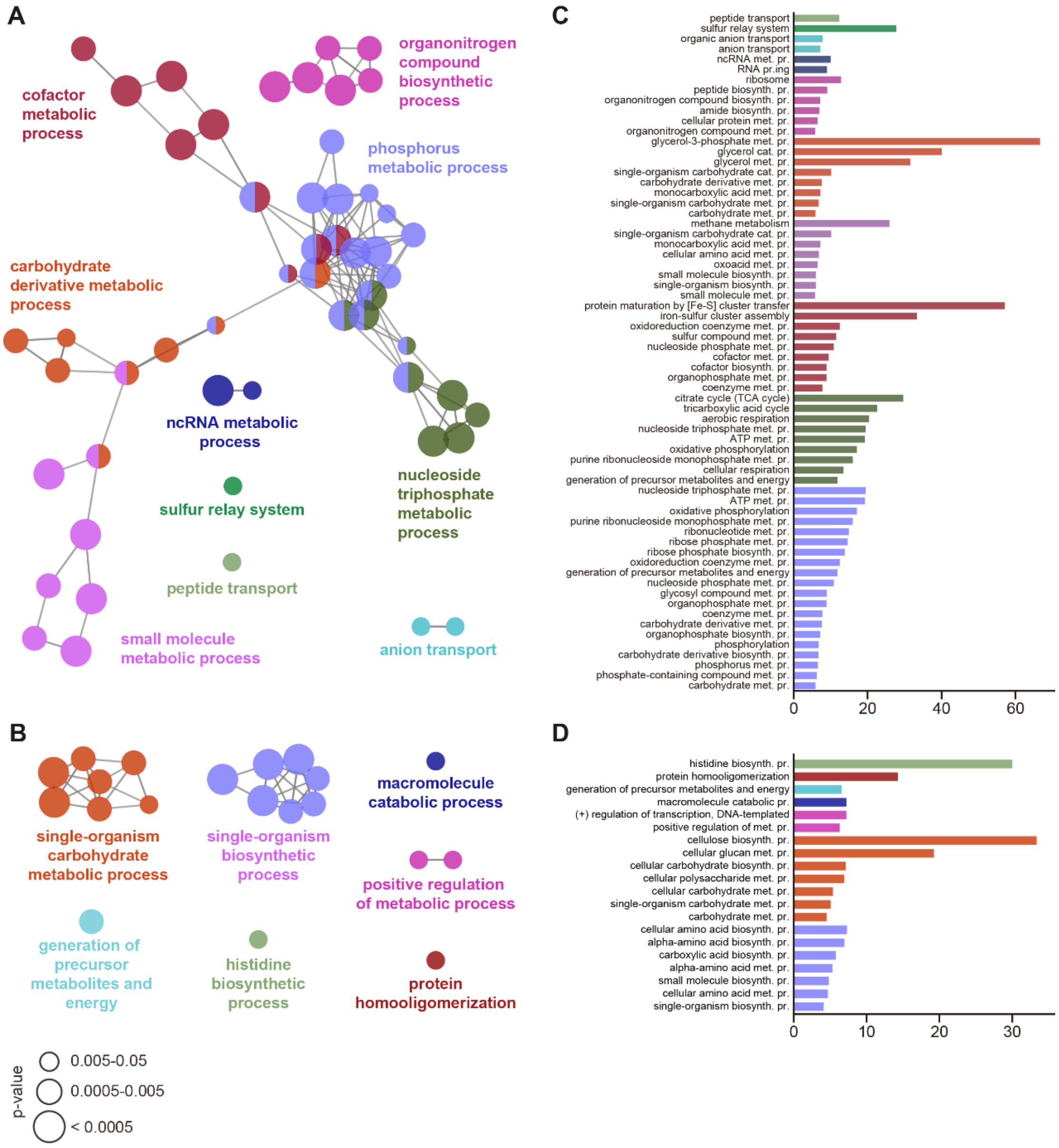

Supplementary Fig. 12. Functional enrichment analysis of DEGs. (A) Network shows enriched pathways and processes in upregulated genes in eMS57. Large number of genes related to cofactor metabolic process, sulfur relay system, small molecule metabolic process, and anion transport were upregulated in eMS57. These genes were responsible for sulfur assimilation to generate Fe-S cluster, S-adenosylmethionine (SAM), and coenzyme A (CoA). In eMS57, a large amount of simple carbon metabolites (such as pyruvate and glyceraldehyde 3-phosphate) enters the lower glycolytic pathway and the TCA cycle. To operate the pathways, a corresponding amount of CoA reservoir would be required because CoA directly accepts pyruvate at the initial step in those pathways. At sulfur assimilation, genes from sulfate (supplemented as a magnesium sulfate in M9 medium) transport through cysteine synthesis were all upregulated 11.4-fold on average. Sulfur fixed into cysteine was utilized to synthesize CoA, SAM, and the iron-sulfur cluster. CoA is synthesized from pyruvate through acetohydroxy acid synthase (AHAS). eMS57 has three copies of AHAS. Expression of AHAS III, which is comprised of *ilvI* and *ilvH*, was

upregulated 3.7 and 1.7-fold, respectively. Expression of one other copy of AHAS was unchanged (AHAS I; *ilvN*+*ilvB*) and the last inactive AHAS II (*ilvM* and pseudogene *ilvG*) was out of the scope of interest. 2-acetolactate converted from pyruvate by AHAS III was further processed into pantoate via *ilvCD*, and *panBE* was upregulated 1.6 times on average. Pantoate is condensed with cysteine to generate CoA by *panC*, *dfp*, and *coaADE*. Alternatively, cysteine is used to synthesize SAM by methionine biosynthesis. Expression of 12 enzymes related to SAM biosynthesis were upregulated 12-fold on average. In addition, sulfur is also used to synthesize the iron sulfur cluster that is a critical cofactor of various enzymes, such as oxidoreductases and dehydrogenases. Although Fe-S cluster synthesis is not yet completely understood, enzymes related to ferrous ion, electron, and Fe-S carriers were all upregulated. Overall, sulfur assimilation and synthesis of sulfur-containing small molecules were upregulated in eMS57. This may contribute to eMS57 remodeling by providing a sufficient reservoir of CoA and other metabolites (B) Network shows enriched pathways and processes in downregulated genes in eMS57. (C and D) Percentage of genes found in a functional term in nodes comprising the network. (C) Nodes of the up-regulated network. (D) Nodes of the down-regulated network. *P*-value was corrected by Bonferroni step-down method (ClueGo). pr.: process, met.: metabolic, biosynth.: biosynthetic, cat.: catabolic.

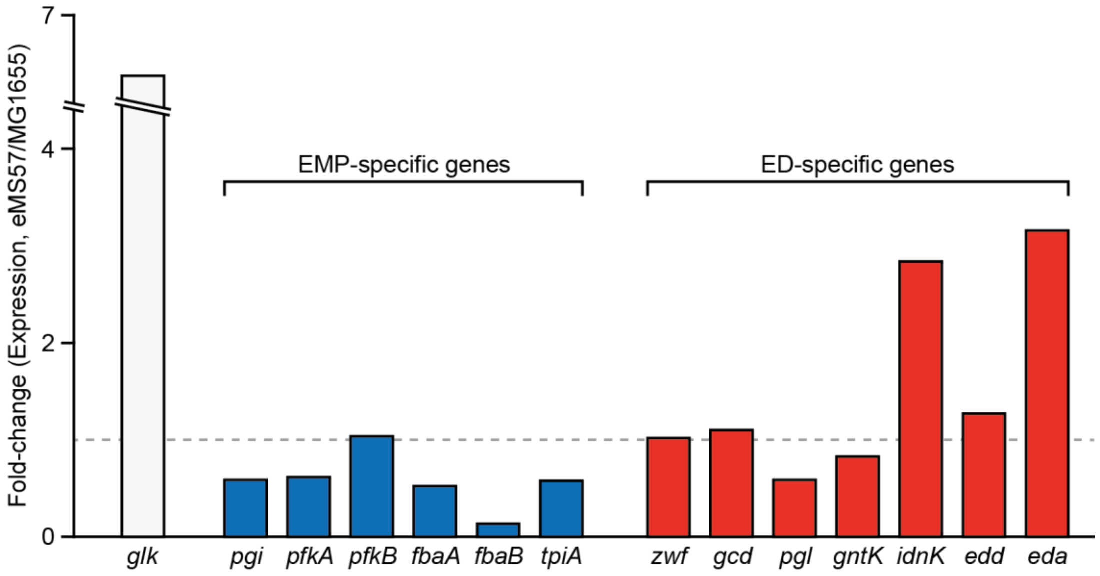

Supplementary Fig. 13. Expression level change of EMP and ED pathways. Gray dotted line indicates fold-change = 1.

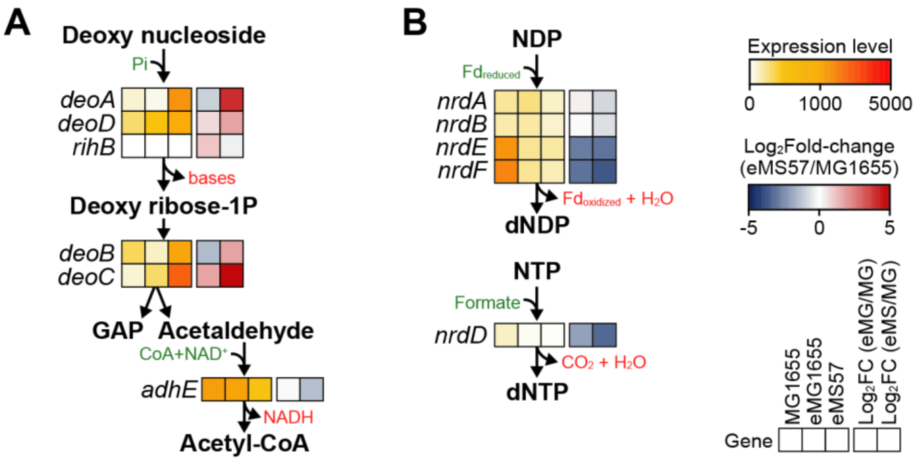

Supplementary Fig. 14. Expression of genes responsible for deoxynucleoside degradation and synthetic pathway. (A) Expression level of deoxynucleoside degradation pathway was increased. (B) Genes related with dNDP/dNTP synthesis from NDP/NTP were down-regulated.

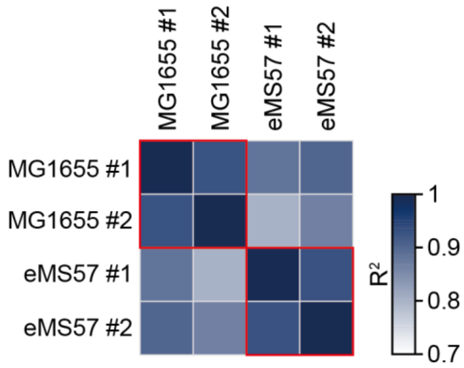

Supplementary Fig. 15. Pearson correlation (r^2) between biological replicates of ribosome profiling. Biological replicates in ribosome profiling correlated with a high correlation constant ($r^2 > 0.93$).

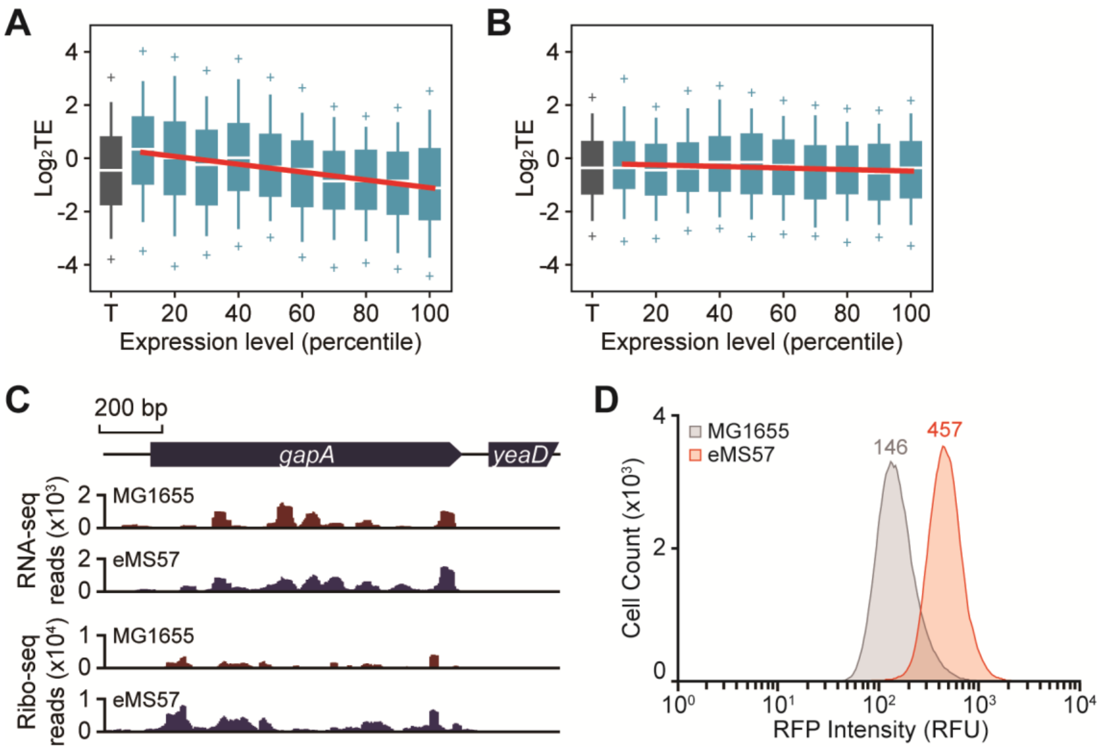

Supplementary Fig. 16. Translational efficiency of MG1655 and eMS57. (A, B) Division of genes in (A) MG1655 or (B) eMS57 into ten bins (percentile) according to their expression level showed translational buffering of genes with high expression level. Translational efficiency equals translation level (RPF) divided by transcription level. T; total genes. Red lines are linear regression of mean values of each bins. Box limits, whiskers, cross marks, center lines indicate 1st and 3rd quartiles, 10 and 90 percentiles, 5 and 95 percentiles, and median of the distribution, respectively. (C) Comparison between RNA-Seq and Ribo-Seq profiles of *gapA* show translational buffering of highly expressed genes. Expression levels are 3484.22 and 3719.73 in MG1655 and eMS57; RPFs are 2223.45 and 3213.62, resulting TE of 0.64 and 0.86 in MG1655 and eMS57, respectively. (D) Flow cytometric measurement of fluorescence protein production revealed that eMS57 produced markedly higher fluorescence. Numbers on the histogram indicate median value of fluorescence intensity.

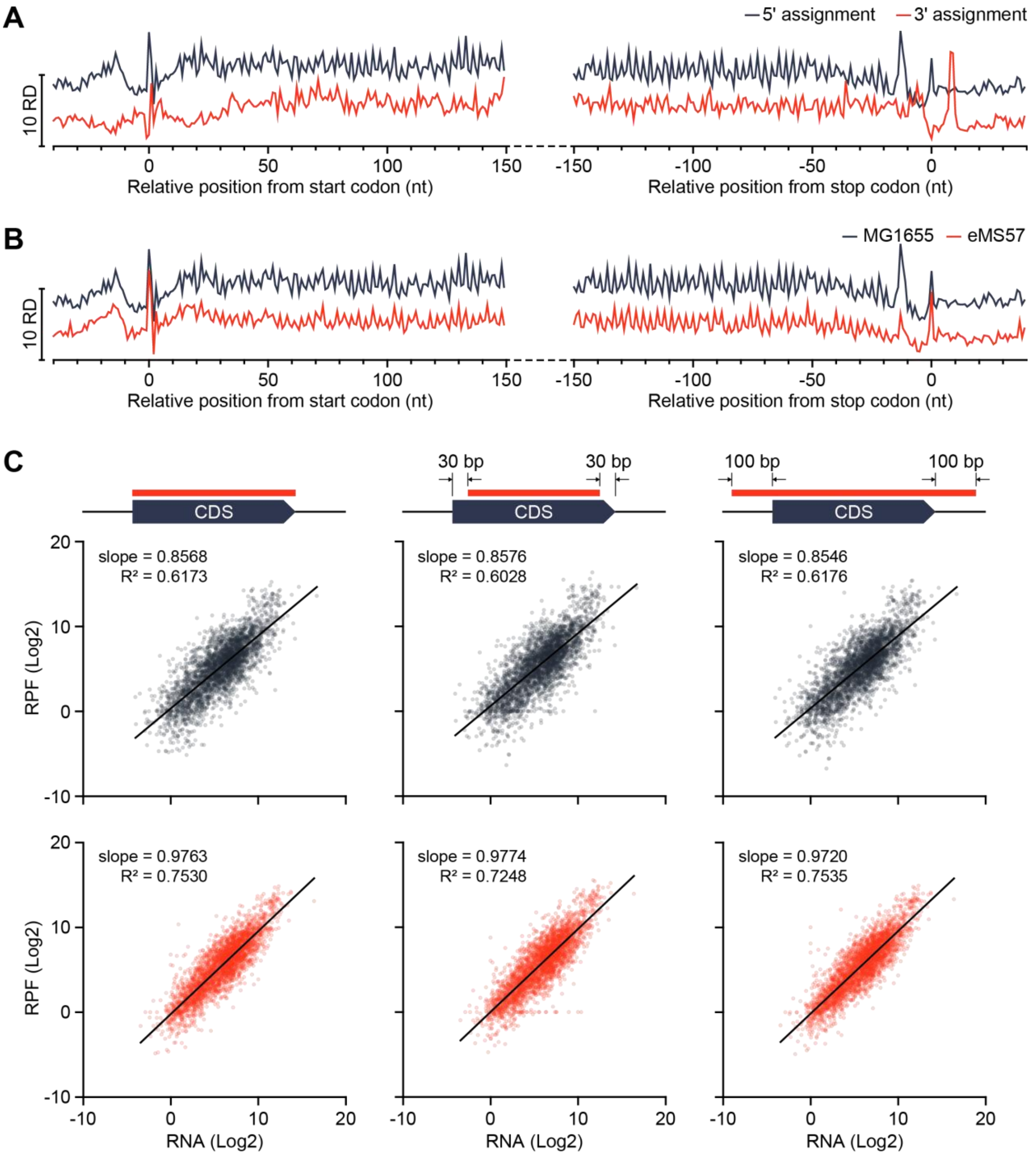

Supplementary Fig. 17. Meta-analysis of Ribo-Seq profile. (A) Average ribosome profile aligned at the start or stop codon by different read assignment method. either 5′ or 3′ ends was tested to determine position of ribosome[2]. 5′ assignment method was used for the meta-analysis, because the method provide clearer 3 nt codon periodicity of translation than 3′ assignment. RD: average normalized ribosome density. Ribosome density was normalized with the maximum peak height in 200 nt window considered. (B) Meta-analysis of ribosome profile on CDSs assigned with 5′ end of the reads. (C) RPF calculated by exact CDS region, CDS excluding

initiation/termination region (30 bp), or CDS including 100 bp upstream/downstream region. Drawings above the graph illustrate the calculated regions.

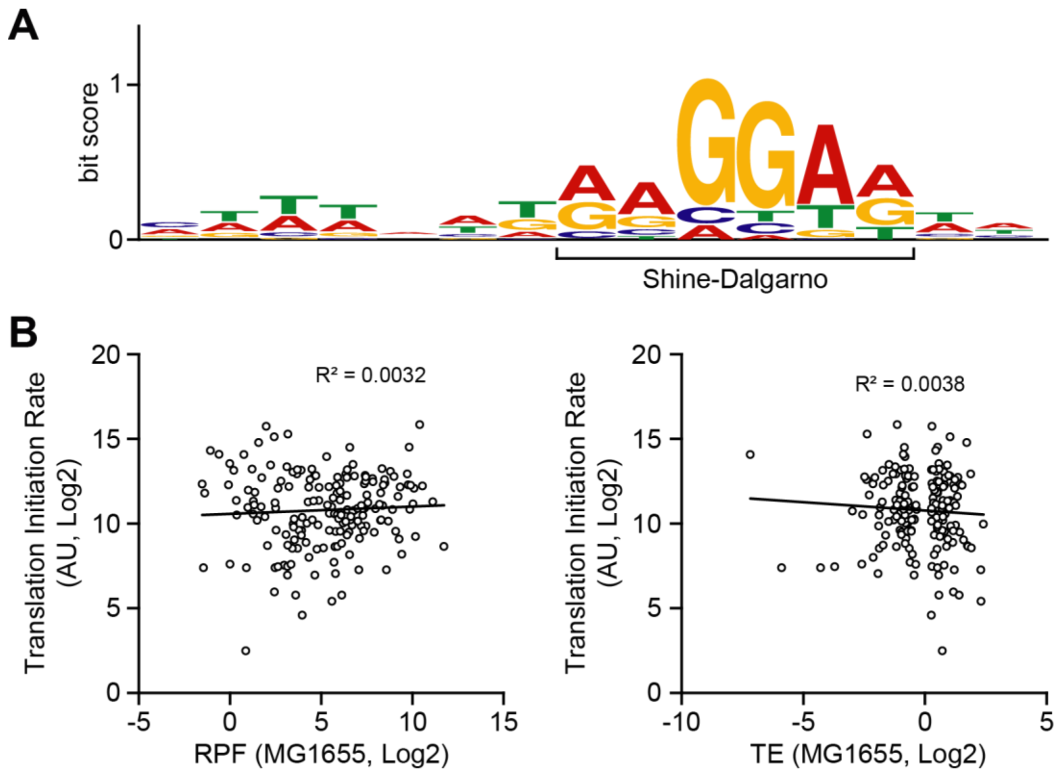

Supplementary Fig. 18. Meta-analysis of sequence motif in 5′ UTR of CDSs. (A) Sequence motif found from 5′ UTR of 91 genes which are translationally buffered in MG1655 and un-buffered in eMS57. (B) Correlation between predicted translation initiation rate and RPF or TE. TIR was calculated from -30 to +30 nt mRNA sequence of the start codon using RBS Calculator[3].

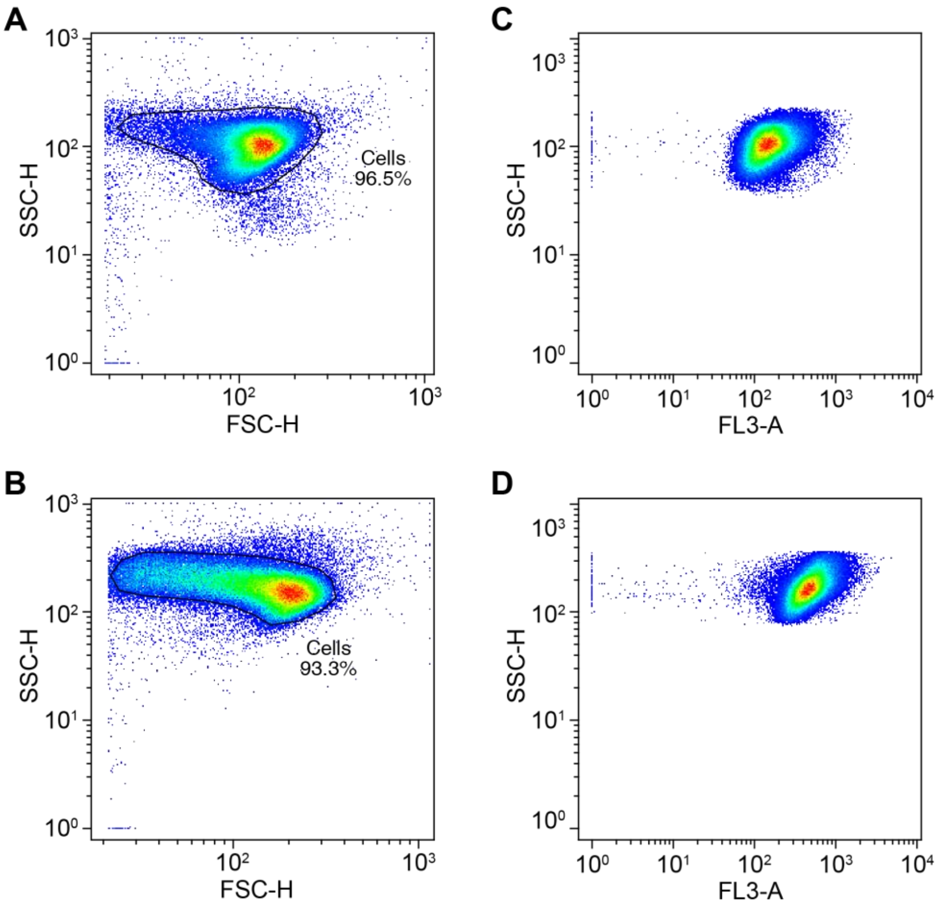

Supplementary Fig. 19. Flow cytometry of MG1655 and eMS57 expressing mRFP. (A) FSC and SSC gating to remove cell debris applied on MG1655. (B) FSC and SSC gating to remove cell debris applied on eMS57. Auto-gating (polygon matching) of ProSort software was used. (C) Contour plot showing red fluorescence intensity in MG1655. (D) Contour plot showing red fluorescence intensity in MG1655.

Supplementary Table 1. Fed-batch fermentation of MG1655 and eMS57. Fermentation was repeated twice in different days. DCW: dried cell weight.

**Medium**	**Strain**	**Biomass (g DCW/L); mean ± s.d.**	**Specific growth rate (h^{-1}); mean ± s.d.**
LB	MG1655	4.917 ± 0.300	1.792 ± 0.017
	eMS57	4.734 ± 0.077	1.240 ± 0.044
M9 glucose	MG1655	1.743 ± 0.098	0.381 ± 0.011
	eMS57	1.671 ± 0.116	0.407 ± 0.004

Supplementary Table 2. Primers used in this study. P: 5′-monophosphate modification.

Experiment	Name	Sequence (5′ to 3′)	
Strain confirmation	large_deletion_F	TTCAGGCCGTAGGTTTTACG	
	large_deletion_R	ACGGGTAGGAGCCACCTTAT	
	hfq_F	GGCTTGACAGTGAAAAACCA	
	hfq_R	TAACCCTCTAAATAGATCAG	
	MDS42_F	GTGGCGAGGCCGTCTATCGC	
	MDS42_R	GCATCTTCGCAAAGCGCGAT	
Heterologous expression of σ^{70}	rpoD_F	GAGGAATAAACCATGGAGCAAAACCCGCAGTC	
	rpoD_R	TTCTGAGATGAGTTTTTGTTCATCGTCCAGGAAGCTACGCA	
	pTrc_inv_F	CTCATCTCAGAAGAGGATCTGGAACAAAAACTCATCTCAGA	
	pTrc_inv_R	GGTTTATTCCTCCTTATTTAATCGATACAT	
21 kb region or *rpoS* knockout	KO_LD_F	GAACCGTAATCCAACACTTAGTGCCCTTCTCCATCGACAGCACGTAGTAAGTGTAGGCTGGAGCTGCTTC	
	KO_LD_R	CTTGGTGAGATTGGTTATTCACCACTGTTAACGGCCGAAGAAGAAGTTTAATTCCGGGGATCCGTCGACC	
	KO_rpoS_F	ATGAGTCAGAATACGCTGAAAGTTCATGATTTAAATGAAGATGCGGAATTGTGTAGGCTGGAGCTGCTTC	
	KO_rpoS_R	TTACTCGCGGAACAGCGCTTCGATATTCAGCCCCTGCGTTTGCAGGATTTATTCCGGGGATCCGTCGACC	
	KO_rpoS_con_F	TTT GCT TGA ATG TTC CGT CA	
	KO_rpoS_con_R	TGA GAC TGG CCT TTC TGA CA	
Amplification primers and TaqMan probes for dPCR	ampD_F	GTCCAGTATGTTCCTTTCGATAAACGT	
	ampD_R	CGTTCGCGCCCCTGATA	
	ampD_reporter_1	TGCGGGAGTCTCTCAG	Dye: VIC, quencher: NFQ
	ampD_reporter_2	CGGGAGCCTCTCAG	Dye: FAM, quencher: NFQ
	ilvN_F	TGCACTTTCACGACATCTTCCA	
	ilvN_R	CCAGCGTCTGGAGCAGAT	
	ilvN_reporter_1	CTTATCGATTTGGCTTATC	Dye: VIC, quencher: NFQ
	ilvN_reporter_2	CTTATCGATTTAGCTTATC	Dye: FAM, quencher: NFQ
	cspC_F	CCGGAGTAATGAAGCCAAAACCTT	
	cspC _R	CAAATGGCAAAGATTAAAGGTCAGGTT	
	cspC_reporter_1	AAGTGGTTCAACGAGTCTA	Dye: VIC, quencher: NFQ
	cspC_reporter_2	AGTGGTTCAACAAGTCTA	Dye: FAM, quencher: NFQ
	yifB_F	CCGGCACTACCGTTTTACTCAA	
	yifB _R	GACCGCTTCGATCTCTCACT	
	yifB _reporter_1	CACCCCCCGGCATT	Dye: VIC, quencher: NFQ
	yifB _reporter_2	CCCCCCCGGCATT	Dye: FAM, quencher: NFQ

SNV reverse engineering	cspC_kan_F	GCTTTATAGCGAGATTGAAGCGTATTCACACTTCAG ATCAGTGGATTCGAGTGTAGGCTGGAGCTGCTTC	
	cspC_kan_R	AGCTATCTGACTGTCAAACATGAGAATTAA	
	cspC_SNP_F	TTAATTCTCATGTTTGACAGTCAGATAGCTGTTACG TTAA	
	cspC_SNP_R	TATCTATGTTATGCCTGCGG	
	cspC_con_F	CACGTTGACGTTGAATACC	
	cspC_con_R	GCATTCGACGAGGCTTTATA	
	ilvN_kan_F	ATACGCGCTTACCTTAACGATAAGCGCGATGTTGTT CAAGCCTTGAGCGGGTGTAGGCTGGAGCTGCTTC	
	ilvN_kan_R	TTTTCAGTAACTGTCAAACATGAGAATTAA	
	ilvN_SNP_F	TTAATTCTCATGTTTGACAGTTACTGAAAAAACACC GCGA	
	ilvN_SNP_R	TCTTTGTCTGCCGATTCAGG	
	ilvN_con_F	GCGCAAAGCCGGAGCGGACG	
	ilvN_con_R	TCAGCAACAGAGTCTGTTCT	
	yifB_kan_F	AAGACTACACCGATTCTGACGATTAATAAAAAGGG CGAAATGCCCTTTTTGTGTAGGCTGGAGCTGCTTC	
	yifB_kan_R	ACTGACATAACTGTCAAACATGAGAATTAA	
	yifB_SNP_F	TTAATTCTCATGTTTGACAGTTATGTCAGTAGTTTCT GCA	
	yifB_SNP_R	ACAGACATTACGTTATCTCA	
	yifB_con_F	AAGACTACACCGATTCTGAC	
	yifB_con_R	GATTGAATCCGGGCAGATCC	
MutS knock-in	mutS_Tn_F	GCATATTGGCTCGAATTCCAACCGATACAATTTTGC GT	
	mutS_neo_R	TAATAAGGGGATCTTTTACACCAGGCTCTTCAAGC	
	mutS_neo_F	AAGAGCCTGGTGTAAAAGATCCCCTTATTAGAAGA	
	neo_Tn_R	CTGCAGGTCGACTCTAGAAGAGCGCTTTTGAAGCTC AC	
	ME Plus 9 – 3’	*P*-CTGTCTCTTATACACATCTCAACCATCA	
	ME Plus 9 – 5’	*P*-CTGTCTCTTATACACATCTCAACCCTGA	
	Tn_confirm_F	GACGGGACGGCGGCTTTGTTGAATA	
	random_R1	CTCGGCATTCCTGCTGAACCGCTCTTCCGATCTNNN NNNNNGCTGG	
qPCR for ChIP confirmation	xseA_F	GGCGGGTTACTATCGACTGA	Amplicon size: 115 bp
	xseA_R	GACGAACCGTTTGATTCAGG	
	yidX_F	GCGCTAAACAGGAGGGAAAG	Amplicon size: 112 bp
	yidX_R	GGAATAAACCGGCAGCCTTA	
	xanP_F	AAAATGCCCGCTTTGCTACC	Amplicon size: 98 bp
	xanP_R	TTCGCTGTTTTGAGTCTGCG	
	purE_F	AACTGCATGGTAGCCCAGTC	Amplicon size: 106 bp
	purE_R	GAGAGTTGTGCACCACAGGA	
	hdeD_F	CTGCATTGCTCACAACTGGC	Amplicon size: 103 bp
	hdeD_R	TCGTGACGGCTCTTTCACTT	

	rsmE_F	TATCCGGTTACCGACTGGGA	Amplicon size: 109 bp
	rsmE_R	TAGGTTAGCTCTTTCGCGCC	
	hpt_F	GCTTTGTTGCGGTGCCAAAA	Amplicon size: 103 bp
	hpt_R	CCGCTTCGGGGATCATTACT	
	nrdR_F	GGGTAAAAGCAGCGAAAGCA	Amplicon size: 104 bp
	nrdR_R	CCTGACCAGGTGGTTAACGG	
	yebS_F	AAACTGGTTTCTCCCGCCAT	Amplicon size: 110 bp
	yebS_R	GGCTGGCAGATCCAGGTAAG	
	yecD_F	AATTCTGCCGCACCGTAAGA	Amplicon size: 109 bp
	yecD_R	TCGGGCAACACCTTTATAGCA	
	glyQ_F	CGTGCTGGATACGTGTGGAT	Amplicon size: 97 bp
	glyQ_R	CATGCGTTAAGCCCTGCTTT	
	bioB_F	GGCAAGATCGTCCGTTGTCA	Amplicon size: 110 bp
	bioB_R	CATGGGGCTTCTCCAAAACG	
	cspE_F	ACTGGTAACCGACACAGCAT	Amplicon size: 109 bp
	cspE_R	GAAACCGAATCCTTTGGACTCA	
	ydiH_F	CGCTTTTAGACAAGACCTGCAC	Amplicon size: 105 bp
	ydiH_R	CCGCCATTTTGTGACGTTGT	
	hybO_F	GGCGCTAAAACGAAGGGAAG	Amplicon size: 95 bp
	hybO_R	TTTATGGCCGGTTATCGCCT	
qRT-PCR for RNA-Seq validation	rcsA_F	AAACCGTTGATGACCTTGCC	Amplicon size: 111 bp
	rcsA_R	TGAGCTTGATACGCTGACTG	
	ydjN_F	ACGCTTTTGTTTACCACGCT	Amplicon size: 101 bp
	ydjN_R	GTTTCTGCACCACCCTGAAC	
	pepN_F	CCGTCATGGTGCATCAGATG	Amplicon size: 105 bp
	pepN_R	TCTTCTTCTTTCCAGGCGGT	
	zapA_F	TGCCGCATTGAATATCAGCT	Amplicon size: 108 bp
	zapA_R	TGTTCTATGGTCTGCTGCAG	
	rplR_F	GCTTTACCCACAGCTGCAG	Amplicon size: 107 bp
	rplR_R	CCGAACGGTTCTGAAGTTCT	
	gadX_F	TAATGCCTCCTCCTTGAGCA	Amplicon size: 104 bp
	gadX_R	CGGATGCAAGCCAAATTCGA	

Supplementary Table 3. Bacterial strains and plasmids used in this study.

**Strain**	**Description**	**Reference**
MG1655	Laboratory *E. coli*, train K-12, substr. MG1655	-
eMG1655	*E. coli* MG1655 adaptively evolved in M9 glucose medium	This study
MS56	*E. coli* MG1655 with large deletions MD1 to MD56	[4]
eMS57	*E. coli* MS56 adaptively evolved in M9 glucose medium	This study
eMS57mutS^{+}	eMS57, *puuP::mutS-kan*	This study
MS56 *Δ21 kb*	MS56, (*hycEDCBA-hypABCDE-fhlA-ygbA-mutS-pphB-ygbIJKLMN-rpoS*)::*kan*	This study
MS56 *ΔrpoS*	MS56, *rpoS::kan*	This study
cspC^{WT}	MS56, *cspC::cspC-kan^{R}*	This study
cspC^{mut}	MS56, *cspC::cspC*(G37A)-*kan^{R}*	This study
ilvN^{WT}	MS56, *ilvN::ilvN-kan^{R}*	This study
ilvN^{mut}	MS56, *ilvN::ilvN*(C202T)-*kan^{R}*	This study
yifB^{WT}	MS56, *yifB::yifB-kan^{R}*	This study
yifB^{mut}	MS56, *yifB::yifB*(1169C insertion)-*kan^{R}*	This study
**Plasmid**	**Description**	**Note**
pKD46	lambda Red (*exo*, *bet*, *gam*), *amp^{R}*, repA101ts ori	[5]
pKD13	FRT-*kanR*-FRT, *amp^{R}*, R6K ori	[5]
BBa_J04450-pSB1C3	BBa_R0010(P_{LacI})-BBa_B0034 (RBS)-BBa_E1010 (*mrfp1*)-BBa_B0015 (terminator), *cm^{R}*, pMB1 ori	[6]

Supplementary References

1 Bindal, G., Krishnamurthi, R., Seshasayee, A. S. N. & Rath, D. CRISPR-Cas-Mediated Gene Silencing Reveals RacR To Be a Negative Regulator of YdaS and YdaT Toxins in *Escherichia coli* K-12. *mSphere* **2** (2017).

2 Woolstenhulme, C. J., Guydosh, N. R., Green, R. & Buskirk, A. R. High-precision analysis of translational pausing by ribosome profiling in bacteria lacking EFP. *Cell Rep.* **11**, 13-21 (2015).

3 Salis, H. M., Mirsky, E. A. & Voigt, C. A. Automated design of synthetic ribosome binding sites to control protein expression. *Nat. Biotechnol.* **27**, 946-950 (2009).

4 Park, M. K. *et al.* Enhancing recombinant protein production with an *Escherichia coli* host strain lacking insertion sequences. *Appl. Microbiol. Biotechnol.* **98**, 6701-6713 (2014).

5 Datsenko, K. A. & Wanner, B. L. One-step inactivation of chromosomal genes in *Escherichia coli* K-12 using PCR products. *Proc. Natl. Acad. Sci.* **97**, 6640-6645 (2000).

6 Campbell, R. E. *et al.* A monomeric red fluorescent protein. *Proc. Natl. Acad. Sci.* **99**, 7877-7882 (2002).
