# Supplementary material for: Adaptive laboratory evolution of a genome-reduced Escherichia coli
Source: Nat Commun. 2019 Feb 25;10:935. doi: 10.1038/s41467-019-08888-6 (PMC6389913; doi:10.1038/s41467-019-08888-6)
Supplement: Supplementary file 3 — Description of Additional Supplementary Files [file 41467_2019_8888_MOESM3_ESM.pdf]

Title: Supplementary Data 1.
Description: Final readout of phenotype microarray.

Title: Supplementary Data 2.
Description: Sequence variations detected by genome re-sequencing.

Title: Supplementary Data 3.
Description: Sequence variations detected by genome re-sequencing of MG1655 ALE experiment.

Title: Supplementary Data 4.
Description: Sequence variations detected by genome re-sequencing of 300 generations of additional ALE experiment.

Title: Supplementary Data 5.
Description: Peak detected from ChIP-seq. UTR, un-translated region. S: shared. D: deleted in MS56. M: MG1655-specific. E: eMS57-specific.

Title: Supplementary Data 6.
Description: Expression level of revealed by RNA-Seq. Statistical significance was tested by Welch's t-test. RPKM; reads per kilobase per million mapped reads.

Title: Supplementary Data 7.
Description: Translation level (RPF) revealed by Ribo-Seq. Translational efficiency is defined as translation level divided by transcription level. RPKM; Reads per kilobase per million mapped reads.
