# Supplementary material for: Adaptive laboratory evolution of a genome-reduced Escherichia coli
Source: Nat Commun. 2019 Feb 25;10:935. doi: 10.1038/s41467-019-08888-6 (PMC6389913; doi:10.1038/s41467-019-08888-6)
Supplement: Supplementary file 11 — Reporting Summary [file 41467_2019_8888_MOESM11_ESM.pdf]

nature research

Corresponding author(s): Byung-Kwan Cho

Last updated by author(s): Jan 29, 2019

Reporting Summary

Nature Research wishes to improve the reproducibility of the work that we publish. This form provides structure for consistency and transparency in reporting. For further information on Nature Research policies, see Authors & Referees and the Editorial Policy Checklist.

Statistics

For all statistical analyses, confirm that the following items are present in the figure legend, table legend, main text, or Methods section.

n/a	Confirmed	
☐	☒	The exact sample size (*n*) for each experimental group/condition, given as a discrete number and unit of measurement
☐	☒	A statement on whether measurements were taken from distinct samples or whether the same sample was measured repeatedly
☐	☒	The statistical test(s) used AND whether they are one- or two-sided<br>*Only common tests should be described solely by name; describe more complex techniques in the Methods section.*
☐	☒	A description of all covariates tested
☐	☒	A description of any assumptions or corrections, such as tests of normality and adjustment for multiple comparisons
☐	☒	A full description of the statistical parameters including central tendency (e.g. means) or other basic estimates (e.g. regression coefficient) AND variation (e.g. standard deviation) or associated estimates of uncertainty (e.g. confidence intervals)
☐	☒	For null hypothesis testing, the test statistic (e.g. *F*, *t*, *r*) with confidence intervals, effect sizes, degrees of freedom and *P* value noted<br>*Give P values as exact values whenever suitable.*
☐	☒	For Bayesian analysis, information on the choice of priors and Markov chain Monte Carlo settings
☐	☒	For hierarchical and complex designs, identification of the appropriate level for tests and full reporting of outcomes
☐	☒	Estimates of effect sizes (e.g. Cohen's *d*, Pearson's *r*), indicating how they were calculated

Our web collection on statistics for biologists contains articles on many of the points above.

Software and code

Policy information about availability of computer code

Data collection	No software was used
Data analysis	Sequencing data was processed on CLC Genomics Workbench v6.5.1 (CLC Bio), sequence motif was analyzed by MEME suite (v4.11.1), and ChIP-Seq peak was called by MACS v1.4.2

For manuscripts utilizing custom algorithms or software that are central to the research but not yet described in published literature, software must be made available to editors/reviewers. We strongly encourage code deposition in a community repository (e.g. GitHub). See the Nature Research guidelines for submitting code & software for further information.

Data

Policy information about availability of data

All manuscripts must include a data availability statement. This statement should provide the following information, where applicable:

- Accession codes, unique identifiers, or web links for publicly available datasets
- A list of figures that have associated raw data
- A description of any restrictions on data availability

Next-generation sequencing data generated during the current study are available in the EMBL Nucleotide Sequence Database (ENA) with accession number PRJEB21199)

Field-specific reporting

Please select the one below that is the best fit for your research. If you are not sure, read the appropriate sections before making your selection.

☒ Life sciences ☐ Behavioural & social sciences ☐ Ecological, evolutionary & environmental sciences

For a reference copy of the document with all sections, see nature.com/documents/nr-reporting-summary-flat.pdf

Life sciences study design

All studies must disclose on these points even when the disclosure is negative.

Sample size	There is no sampling (or sub-sampling) done in this study. More than two replicates were generated and all the generated samples were analyzed.
Data exclusions	Not applicable in this study.
Replication	All experiments were replicated with more than two biological replicates. Reproducibility of data was analyzed by calculating Pearson's correlation constant. Please see Supplementary Information for individual correlation coefficients and reproducibility description.
Randomization	Not applicable in this study.
Blinding	Not applicable in this study.

Reporting for specific materials, systems and methods

We require information from authors about some types of materials, experimental systems and methods used in many studies. Here, indicate whether each material, system or method listed is relevant to your study. If you are not sure if a list item applies to your research, read the appropriate section before selecting a response.

Materials & experimental systems

n/a	Involved in the study	
☐	☒	Antibodies
☒	☐	Eukaryotic cell lines
☒	☐	Palaeontology
☒	☐	Animals and other organisms
☒	☐	Human research participants
☒	☐	Clinical data

Methods

n/a	Involved in the study	
☐	☒	ChIP-seq
☐	☒	Flow cytometry
☒	☐	MRI-based neuroimaging

Antibodies

Antibodies used	1. Anti-RpoD mouse-IgG; NeoClone; Clone 2G10; Cat. #663202; Lot. #B193929<br>2. Anti-MutS rabbit polyclonal; GeneCheck; Cat. #GC-M001; Lot. #219<br>3. Anti-c-Myc Mouse-IgG (Santa Cruz Biotechnology; Clone 9E10; Cat. #sc-40; Lot. #B1313)<br>4. Normal Mouse IgG (Millipore, Cat. #12-371, Lot. #DAM1774722)
Validation	Cross-reactivity of monoclonal antibody in E. coli was tested by manufacterer (https://www.biolegend.com/en-us/products/purified-anti-eme--coli-em-rna-sigma-70-antibody-10486). Also cross-reactivity of antibodies was confirmed by WB and real-time PCR before ChIP-Seq experiments.

ChIP-seq

Data deposition

☒ Confirm that both raw and final processed data have been deposited in a public database such as GEO.

☒ Confirm that you have deposited or provided access to graph files (e.g. BED files) for the called peaks.

Data access links<br>*May remain private before publication.*	ChIP-Seq sequencing data is available in the European Nucleotide Archive (ENA) by EMBL-EBI with accession number PRJEB21199 [https://www.ebi.ac.uk/ena/data/view/PRJEB23980].
Files in database submission	In PRJEB21199 project, individual sequence data of ChIP-Seq is available with accession numbers of ERS1779907, ERS1779906, ERS1779905, ERS1779904, ERS1779903, and ERS1779902

Genome browser session (e.g. UCSC)	No longer applicable.

Methodology

Replicates	ChIP-Seq experiments were done with two biological replicates and one mock sample per strain. Mock samples were generated by treating a normal mouse IgG antibody instead of a specific primary antibody.
Sequencing depth	Sequencing was done with read length of 50 bp and single-ended recipe.<br>1. MG1655 mock sample: 1,458,692 reads were mapped uniquely out of 1,523,821 raw reads. Covers 15.90-fold genome.<br>2. MG1655 SigD ChIP-Seq replicate #1 sample: 1,460,536 reads were mapped uniquely out of 1,494,140 raw reads. Covers 15.91-fold genome.<br>3. MG1655 SigD ChIP-Seq replicate #2 sample: 1,783,995 reads were mapped uniquely out of 1,816,635 raw reads. Covers 19.44-fold genome.<br>4. eMS57 mock sample: 2,069,834 reads were mapped uniquely out of 2,275,676 raw reads. Covers 22.58-fold genome.<br>5. eMS57 SigD ChIP-Seq replicate #1 sample: 2,294,524 reads were mapped uniquely out of 2,357,189 raw reads. Covers 25.01-fold genome.<br>6. eMS57 SigD ChIP-Seq replicate #2 sample: 2,582,956 reads were mapped uniquely out of 2,685,537 raw reads. Covers 28.16-fold genome.
Antibodies	1. Anti-RpoD mouse-IgG; NeoClone; Clone 2G10; Cat. #663202; Lot. #B193929<br>2. Anti-c-Myc Mouse-IgG (Santa Cruz Biotechnology; Clone 9E10; Cat. #sc-40; Lot. #B1313)<br>3. Normal Mouse IgG (Millipore, Cat. #12-371, Lot. #DAM1774722)
Peak calling parameters	1. Read mapping was done in CLC Genomics Workbench v6.5.1 (CLC Bio; mismatch cost: 2, indel cost: 3, length and similarity fractions: 0.9) and mapping file was exported as a bam file.<br>2. The bam files were submitted to MACS v1.4.2 software. Peaks were called with following parameters:<br>- Control file: mock mapping file.bam<br>- Effective genome size: 4.64 x 10^6<br>- Band width: 300<br>- Model fold: 10, 30<br>- p-value cutoff: 1.00e-5<br>- Large dataset will be scaled towards smaller dataset.<br>- Range for calculating regional lambda is: 1000 bps and 10000 bps<br>- Tag size: auto
Data quality	All the peaks detected had FDR less than 0.12%. 58.1% of detected peaks had fold-enrichment over 5, and 96% of the peaks have fold-enrichment over 3.
Software	The binding peak was called using MACS software (v1.4.2)

Flow Cytometry

Plots

Confirm that:

☒ The axis labels state the marker and fluorochrome used (e.g. CD4-FITC).

☒ The axis scales are clearly visible. Include numbers along axes only for bottom left plot of group (a 'group' is an analysis of identical markers).

☒ All plots are contour plots with outliers or pseudocolor plots.

☒ A numerical value for number of cells or percentage (with statistics) is provided.

Methodology

Sample preparation	Cells harboring high copy mRFP1 expression plasmid, BBa_J04450-pSB1C3, were grown aerobically in M9 glucose medium for 12 h at 37 °C, with 0.5 mM of IPTG induction. Then, 1 ml of the cell culture was diluted in 9 ml of PBS and cells were dissociated using a round bottom polystyrene test tube with cell strainer snap cap (Corning)
Instrument	S3e Cell Sorter (Bio-Rad)
Software	Instrument running and data collection was done by ProSort software (Bio-Rad). Data analysis was done on FlowJo software (v10, FlowJo).
Cell population abundance	Not applicable in this study, cell sorting was not conducted.
Gating strategy	Gating was applied on FCS-SSC plot to avoid any non-cell debris. Auto-gating (polygon matching) of ProSort software was used. After gating, 96.5 (MG1655) and 93.3% (eMS57) of event remained. Advanced gating strategy is not applicable in this study, because it is not associated with any staining or biomarker.

☒ Tick this box to confirm that a figure exemplifying the gating strategy is provided in the Supplementary Information.
